# Interspecies interactions among sugarcane-associated bacteria and their impact on plant growth promotion traits

**DOI:** 10.3389/fmicb.2026.1714037

**Published:** 2026-01-29

**Authors:** César Justiniano Fascio, Anna Carolina Rubio Molina, Benyi Juliana Marin-Gallego, Ricardo Ezequiel de Cristóbal, Manuel Espinosa-Urgel, Paula Andrea Vincent, Juan Vicente Farizano, Conrado Adler

**Affiliations:** 1 Instituto Superior de Investigaciones Biológicas (INSIBIO), CONICET-UNT and Instituto de Química Biológica “Dr. Bernabé Bloj”, Facultad de Bioquímica, Química y Farmacia, UNT, San Miguel de Tucumán, Tucumán, Argentina; 2 Department of Biotechnology and Environmental Protection, Estación Experimental del Zaidín, CSIC, Granada, Spain

**Keywords:** bacterial interactions, bacterial isolation, PGP traits, rhizosphere and endophytic bacteria, sugarcane

## Abstract

Microbes associated with plants have proven to play a fundamental role in their growth and phytosanitary status. Microbial community architecture and function results from interactions with the host and with each other. Therefore, microbial diversity and the array of possible interspecies interactions should be considered as key elements for the development of future biocontrol and crop improvement strategies. To gain some insight into this potential, we isolated 16 rhizospheric and 16 endophytic bacteria from sugarcane and tested their ability to interact with each other. To this end, we performed 120 pairwise interaction assays within each group. Although most interactions were neutral in both rhizospheric and endophytic communities, negative interactions were more frequent between rhizospheric isolates. In contrast, positive ones predominated among endophytic isolates. After determining the interaction phenotypes between isolates, we tested their impact on plant growth promoting (PGP) traits and biocontrol against *Xanthomonas albilineans*. Our results demonstrate that interspecies interactions among sugarcane-associated bacteria can modulate key PGP traits regardless of their interaction phenotype, highlighting a potentially overlooked layer of functional regulation within the microbiome. Accordingly, social behavior of microorganisms might set the basis for a rational design of performance-improved bioinoculants for agriculture, particularly consortia-based inoculants.

## Introduction

Interaction between plants and their associated microbial communities play a crucial role in plant development, health, and productivity (Trivedi et al., 2020). These communities contribute to key ecological processes such as nutrient cycling, disease suppression, and stress mitigation (Berendsen et al., 2012). Among them, plant growth-promoting bacteria (PGPB) enhance plant fitness through mechanisms including phosphate solubilization, siderophore production, and phytohormone synthesis, particularly indole-3-acetic acid (IAA; Trivedi et al., 2020; Chen et al., 2024). While these beneficial effects are often attributed to individual strains, there is increasing recognition that microbial functions emerge from complex interspecies interactions within the microbial community (Trivedi et al., 2020). Sugarcane (*Saccharum officinarum*), a globally important crop, is frequently cultivated through ratooning and long-term monoculture (Xu et al., 2021). In some regions, this practice has persisted for decades, creating a unique agroecosystem with a growing selective pressure on the associated microbiota (Pang et al., 2021; Khan et al., 2023). Over time, these conditions lead to the selection of microbial communities and significant changes in the physicochemical properties of soil (Pang et al., 2021). Previous studies have shown that these microbes evolve in response to specific root exudates and environmental factors, forming functionally specialized consortia (Xun et al., 2021; Tayyab et al., 2022). This characteristic environment makes sugarcane-associated microbes ideal to study microbial interspecies interactions. Despite advances in the characterization of sugarcane-associated microbiomes, there is limited understanding of how microbial interactions influence PGP traits. Microbial interactions can modulate gene expression and metabolite production, thereby affecting their functional output (Tyc et al., 2014; Grandchamp et al., 2017). These interactions may enhance or suppress beneficial traits like nutrient solubilization or biocontrol capacity, and thus have profound implications for designing microbial inoculants. Indeed, inconsistent field performance of bioinoculants may partly reflect our limited grasp of how intermicrobial dynamics shape trait expression *in situ* (Gu et al., 2020). In this study, we examined the interactions among sugarcane-associated bacteria isolated from the rhizosphere and endosphere of plants cultivated under a 100-year-old monoculture system. Through a systematic analysis of 120 binary combinations within each microbial group, we characterized interaction phenotypes and their effects on key PGP traits, including phosphate solubilization, siderophore and IAA production. Furthermore, we assessed their biocontrol potential against *Xanthomonas albilineans*, a major sugarcane pathogen. By systematically characterizing how pairwise interactions reshape plant-beneficial traits, this work contributes to the emerging effort to predict community-level functional outputs from simple interaction rules, an essential step toward rationally engineering microbial consortia.

## Materials and methods

### Microbial isolation

Sugarcane var pTUC 85-384 plants were randomly selected from a monoculture plot over 100 years old from Las Talitas, Tucumán, Argentina. To obtain a representative composite sample, five plants were pooled. Bacterial isolates were obtained from two distinct plant compartments: the rhizosphere and the endosphere (stem). To isolate rhizospheric bacteria, roots were gently shaken to remove loosely associated soil, and the soil that remained tightly attached to the roots was considered rhizosphere soil. Two grams of this attached rhizospheric soil were resuspended in 4 ml of physiological saline solution. Serial dilutions were plated on LB, LB 0.1X, and M9 medium [supplemented with 0.1% casamino acids, 1 mM MgSO_4_, 1 mg/ml vitamin B1, and 0.2% (w/v) sucrose, glucose, or glycerol as carbon sources] for bacterial isolation. Plates were incubated at 30 °C for 4 days and inspected daily. On the other hand, endophytic bacteria isolation was performed following the protocol described by Aravind et al. (2010) with modifications. Briefly, sugarcane stems were superficially disinfected using sequentially 10% sodium hypochlorite, 70% alcohol and rinsed three times with sterile distilled water. Surface sterility was checked by plating 100 μl of the last wash on LB agar plates and verifying no growth after 7 days at 30 °C. Internal tissue samples (2 g) were crushed aseptically in physiological saline solution (4 ml), and the supernatant was serially diluted and plated on the same media used for rhizospheric bacteria. In this case, plates were incubated for 7 days at 30 °C. We selected rhizospheric (*n* = 16, named RB) and endophytic (*n* = 16, named EB) isolates with similar growth rates (fast growers) and different colony morphology. Isolates were preserved in 20% glycerol at −70 °C. To estimate the microbial abundance in the rhizosphere and endosphere samples, CFU per gram of sample was calculated after measuring CFU/ml obtained by plating serial dilutions of the original suspensions (rhizospheric or endophytic) into LB 0.1X. CFU/g was calculated as the average for triplicates.

### Isolates identification by 16S rRNA gene sequencing and phylogenetic tree

Isolates were taxonomically identified by amplification, sequencing and subsequent 16S rRNA analysis. Colony PCR was followed using universal primers 27F (5′-AGAGTTTGATCMTGGCTCAG-3′) and 1492R (5′-GGTTACCTTGTTACGACTT-3′) to amplify the 16S rRNA. Amplification was performed using an BioRad T100 Thermal Cycler with the following PCR conditions: initial denaturalization 95 °C 5 min, 35 cycles of 94 °C for 30 s, 52 °C for 30 s and 72 °C for 2 min; and a final extension at 72 °C 10 min. PCR products sequencing was carried out in Macrogen Inc. (Seoul, Republic of Korea). Sequences obtained were compared with the nucleotide database available in the BLAST tool at the National Center for Biotechnology Information (NCBI; http://www.ncbi.nlm.nih.gov). The sequences were deposited in the GenBank database under the accession numbers detailed in Table 1. Then, a phylogenetic analysis was carried out where 16S rRNA gene sequences of the different isolates were analyzed using the Alignment, Classification and Tree Service of the SILVA database (https://www.arb-silva.de/aligner; Westram et al., 2011; Chuvochina et al., 2026). Isolates and neighbor sequences were used for pairwise and multiple alignment using MEGA12 (Kumar et al., 2024). The results were used to build a phylogenetic tree, in which the evolutionary history was inferred using the Neighbor-Joining method (Saitou and Nei, 1987), with a bootstrap test of 5,000 replicates (Felsenstein, 1985). The evolutionary distances were computed using the Maximum Composite Likelihood method (Tamura et al., 2004) and are in the units of the number of base substitutions per site. The analytical procedure encompassed 76 nucleotide sequences. The pairwise deletion option was applied to all ambiguous positions for each sequence pair resulting in a final data set comprising 50,000 positions.

**Table 1:** Sugarcane rhizospheric and endophytic isolates.

**Isolates**	**16S identification**	**Accession number**	**Phosphate solubilization (PSI)**	**Siderophore production**	**IAA production**	***X. albilineans* biocontrol**
RB 202	*Variovorax* sp.	PX421667	–	–	–	+
RB 204	*Bacillus* sp.	PX421668	–	++	+	–
RB 208	*Arthrobacter* sp.	PX421669	–	++	–	–
RB 214	*Enterobacter* sp.	PX421670	1.22	++	+	+
RB 216	*Chryseobacterium* sp.	PX421671	–	–	++	+
RB 217	*Pseudomonas* sp.	PX421672	1.20	–	–	–
RB 219	*Curtobacterium* sp.	PX421673	–	–	–	–
RB 241	*Rhizobium* sp.	PX421674	–	++	–	–
RB 258	*Burkholderia* sp.	PX421675	1.40	+	–	+
RB 266	*Herbaspirillum* sp.	PX421676	–	–	–	–
RB 268	*Azospirillum* sp.	PX421677	–	–	–	–
RB 273	*Novosphingobium* sp.	PX421678	–	–	–	–
RB 274	*Microbacterium* sp.	PX421679	–	–	+	–
RB 275	*Sphingomonas* sp.	PX421680	–	–	+	–
RB 277	*Flavobacterium* sp.	PX421681	–	–	–	–
RB 278	*Pedobacter* sp.	PX421682	–	–	–	–
EB 331	*Sphingomonas* sp.	PX421683	–	–	–	–
EB 332	*Acinetobacter* sp.	PX421684	–	–	–	–
EB 333	*Staphylococcus* sp.	PX421685	–	–	++	–
EB 334	*Agrobacterium* sp.	PX421686	–	–	+	–
EB 335	*Rothia* sp.	PX421687	–	–	–	–
EB 336	*Pantoea* sp.	PX421688	1.25	+	+	–
EB 338	*Acinetobacter* sp.	PX421689	–	–	–	–
EB 340	*Pseudomonas* sp.	PX421690	1.10	–	–	–
EB 341	*Sphingobium* sp.	PX421691	–	–	+++	–
EB 342	*Microbacterium* sp.	PX421692	–	–	+	–
EB 343	*Bacillus* sp.	PX421693	–	–	–	–
EB 344	*Kocuria* sp.	PX421694	–	+	–	–
EB 351	*Acinetobacter* sp.	PX421695	–	–	–	–
EB 353	*Acinetobacter* sp.	PX421696	–	–	–	–
EB 354	*Brevundimonas* sp.	PX421697	–	–	–	–
EB 355	*Brevundimonas* sp.	PX421698	–	–	–	–

Siderophore production (halo size, mm): +, 1–5; ++, 5–14; +++, 15–20. IAA production (color intensity, AU): +, 1–5; ++, 5–10; +++, >10.

### Isolates characterization for PGP traits and biocontrol against *X. albilineans*

In order to characterize each strain, we performed *in vitro* assays, such as phosphate solubilization capacity, IAA and siderophore production and the ability to inhibit *in vitro* a sugarcane pathogen, *X. albilineans*.

#### Phosphate solubilization

Phosphate solubilization was determined using solid NBRIP medium [10 g/L glucose, 2.5 g/L Ca_3_(PO_4_); 5 g/L MgCl_2_.6H_2_O, 0.25 g/L MgSO_4_.7H_2_O, 0.2 g/L KCl, and 0.1 g/L (NH_4_)_2_SO_4_], as described previously (Nautiyal, 1999). Bacterial isolates were grown ON in M9 minimal medium supplemented with 0.2% sucrose (M9suc). Then, cells were washed twice with physiological saline solution and aliquots of 5 μl were placed by triplicate in NBRIP solid medium. After 5 days of incubation at 30 °C, the solubilization index was determined (Mohamed et al., 2018). The diameters of the colonies and the halos were measured and the Phosphate Solubilization Index (PSI) for each strain was calculated as the ratio between the diameter of the halo and the diameter of the colony (PSI = D halo/D colony; Mohamed et al., 2018).

#### Siderophore production

The siderophore production was determined by a qualitative technique, adapted in our laboratory, based on the color change of Cromoazurol S. Strains were resuspended in physiological saline solution. Five microliter aliquots of each suspension were seeded on M9suc plates and incubated at 37 °C for 48 h. Then, an overlay of semisolid CAS medium (60.5 mg/L Chrome Azurol S, 72.9 mg/L PIPES, 10 μM FeCl_3_, 0.9% Agar) was poured onto the grown plate. Those isolates that were capable of producing siderophores presented a color change of the CAS medium from blue to yellowish around the colony. Isolates with wider halos (diameter) and higher color change were considered as stronger producers in a semi-quantification approach.

#### IAA production

Determination of total indoles was carried out using the colorimetric method described by Glickmann and Dessaux (1995) with modifications. Strains were resuspended in physiological saline solution and 5 μl aliquots of each isolate were grown in M9suc agar plates at 30 °C for 48 h. Once cells grew in the agar plate, a filter paper soaked in the Salkowski reagent (0.5 M FeCl_3_; 35% HClO_4_) was placed over each plate. Then, plates were incubated for 30 min in the dark at room temperature. After the incubation period, filter papers were carefully extracted and photographed. To estimate relative IAA production (semi-quantitative), color signal quantifications of each filter paper were obtained from the photographs using Trigit (Tjandra et al., 2023). Color extraction and analysis was performed sampling at equal distances (from the border of each colony). Each colony was analyzed within its own region of interest (ROI) considering an equal number of pixels (arbitrarily 2,500). Trigit computes mean color parameters across all pixels inside the ROI; therefore, the colony diameter does not influence the resulting color values. Images were acquired under controlled and consistent illumination to avoid overexposure.

#### Biocontrol activity against sugarcane phytopathogenic bacteria *X. albilineans*

Each isolate was tested for its ability to inhibit the growth of sugarcane pathogen *X. albilineans*, a bacteria responsible for leaf scald disease. The phytopathogen was grown in M9suc liquid medium at 30 °C for 24 h with shaking (160 rpm). Then, cells were washed twice with physiological saline solution and its OD600 nm was adjusted to 0.1. The suspension was seeded with a sterile swab on M9suc-agar plates in order to obtain a cell lawn. Five microliter spots of each isolate suspension were placed by triplicate over the cell lawn and plates were placed for 48 h at 30 °C. The formation of a clear halo around the colony indicated the ability to inhibit pathogen growth.

### Microbial interactions

To reveal communication between the selected microorganisms we performed intermicrobial interaction assays. For this purpose, isolates were placed in M9suc solid medium following a distance pattern generating binary interactions (Supplementary Figure S1A). EB were tested against each other, as well as RB, and all possible pairwise combinations were performed. For this, bacterial suspensions were obtained from colonies at an OD600 nm between 0.2 to 0.3. Three spots of 5 μl of the bacterial suspension were inoculated on M9suc agar plates following the pattern of distances mentioned above (Supplementary Figure S1A). The distal spots were used as a control. Plates were incubated at 30 °C for 48 h. Macroscopically observable interactions were analyzed and classified to summarize all possible pairwise interactions, according to Faust and Raes (2012). Three possible outcomes were considered for each interaction partner: positive (one strain benefits while the other remains unaffected or both partners benefit), negative (one species is harmed while the other is unaffected, or one species benefits at the expense of the other, or both species are negatively affected), and neutral (neither species affects the other; representative interactions are shown in Supplementary Figure S1B). Furthermore, we identified a particular category that we termed *singular*. These are defined by the ability of a single strain to exert a distance-dependent effect on another isolate (Supplementary Figure S1B). Each type of interaction and its frequency was calculated.

### Impact of microbial interactions on PGP properties

To assess whether microbial interactions influence PGP traits, we reproduced the same distance-based interaction assay described above, but performing it directly on the specific media required for each PGP determination. Instead, pairwise interactions were established on solid medium by spotting 5 μl of each bacterial suspension (OD600 nm = 0.2–0.3) following the standardized spatial arrangement shown in Supplementary Figure S1A. After incubation, PGP traits were revealed exactly as described for single-strain assays, using the appropriate detection method for each property. Only isolates that displayed activity in monoculture for a given PGP trait were evaluated against the 15 remaining isolates. Interaction effects were inferred by comparing the PGP signal of each spot with its corresponding monoculture control.

## Results and discussion

### Diversity of sugarcane-associated bacterial isolates

**Figure 1:**
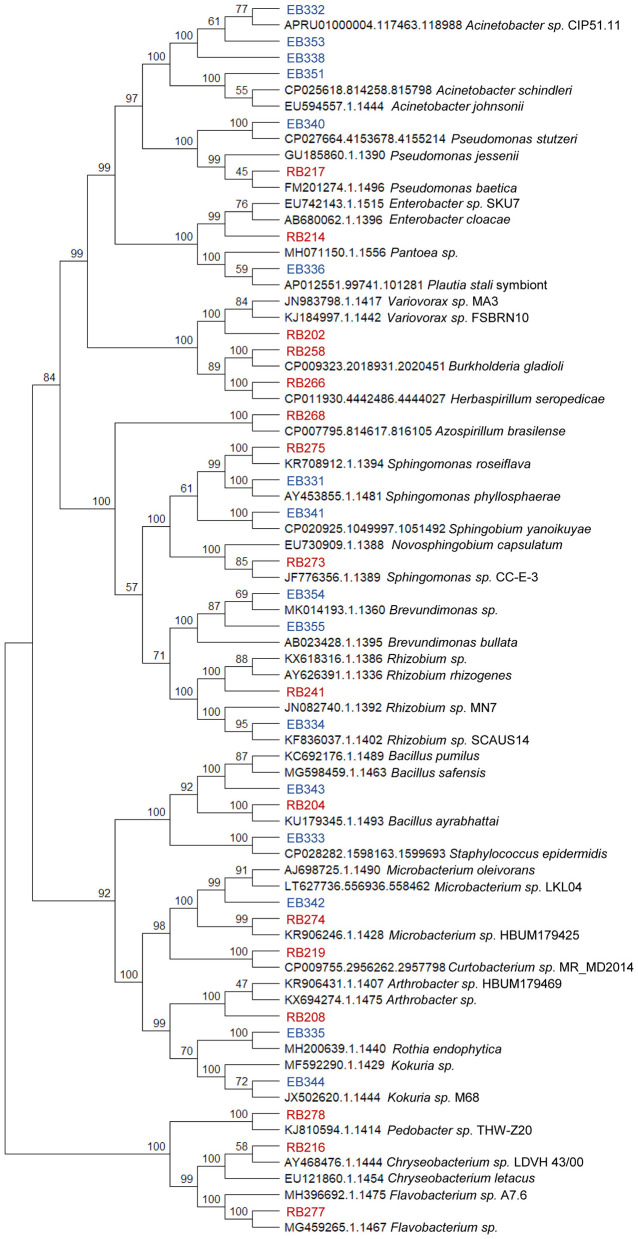
Evolutionary relationships of rhizosphere (RB, red) and endophytic (EB, blue) bacteria isolated from sugarcane and their closest taxa, based on 16S rRNA gene sequences (see text for details). The evolutionary history was inferred using the Neighbor-Joining method (Saitou and Nei, 1987), and the optimal tree with the sum of branch length = 2.020 is presented. The percentage of replicate trees in which the associated taxa clustered together in the bootstrap test (5,000 replicates) are shown above the branches (Felsenstein, 1985). Accession numbers and names correspond to closest relatives in the SILVA database. Evolutionary analyses were conducted in MEGA12 (Kumar et al., 2024) utilizing up to four parallel computing threads.

Sugarcane var pTUC 85-384 plants were used to isolate rhizospheric and stem endophytic bacteria as described in the Materials and methods section. Microbial abundance was measured in terms of CFU per gram of sample, and the results showed a significantly higher abundance in the rhizosphere (1 × 10^5^ CFU/g of soil) compared to the endosphere (1 × 10^2^ CFU/g of stem tissue). The significant difference in bacterial abundance between the rhizosphere and endosphere is in line with previous studies that show that the stem endosphere typically harbors less abundant microbial populations due to a host selection as well as differential exposure to environmental conditions such as greater temperature fluctuations (Hu et al., 2022). A total of 32 bacterial strains were selected for further analysis: 16 rhizospheric bacteria (RB) and 16 endophytic bacteria (EB). The selection was based on differences in macroscopic colony characteristics, such as morphology and color and similitude in growth kinetics (fast growers). The latter was intended to avoid bias in interactions due to differences in cell densities (Yu et al., 2024). Also, the use of fast growers strains ensure practical inoculant potential, as these are more impactful on plant growth and easier to produce at scale (Lopez et al., 2023). Identification of these isolates was carried out using 16S rRNA gene sequencing, which allowed for genus-level classification. All RB isolates were assigned to different genera, underscoring the high taxonomic diversity within this group, while the EB isolates comprised 12 distinct genera (Table 1). A phylogenetic tree (Figure 1) was constructed to visualize the genetic relationships among these isolates. Most of the genera identified in this study have been previously reported as part of bacterial communities within sugarcane rhizosphere and plant tissues respectively (Mendes et al., 2007; de Souza et al., 2016; Armanhi et al., 2017; Pereira et al., 2019). The tree showed that, while some of the identified bacterial groups can be found in both niches (*Bacillus, Rhizobium, Sphingomonas*, and *Microbacterium*), in general there seems to be little overlap, which could be suggestive of niche specialization. It is also worth mentioning the presence of isolates related to genera that include human pathogens, such as *Chryseobacterium* or *Staphylococcus*. Both have been found associated with different plants, including sugarcane, supporting the idea that plants can function as reservoirs for certain pathogens (Magnani et al., 2010; Jayakumar et al., 2020; Zhang et al., 2021). Although members of *Burkholderiaceae*, including genera such as *Herbaspirillum* and *Burkholderia*, are highly abundant in sugarcane tissues (de Souza et al., 2016), endophytes must transit through the rhizosphere during the early steps of plant colonization. Thus, their detection in rhizospheric samples is fully consistent with their ecology. The lack of endophytic representatives of these genera in our study could be due to isolation-related methodological biases such as surface-sterilization and tissue maceration procedures, and plant developmental stage which can strongly influence the recovery of endophytes.

### Functional profiling of sugarcane-associated bacteria

PGP traits are key microbial functions that can enhance plant development, nutrient acquisition, and stress tolerance, making them crucial targets in the selection of beneficial bacterial strains (El-Saadony et al., 2022). The PGP traits of each of the 32 isolates were tested for three key biochemical properties: phosphate solubilization, siderophore production, and IAA production. Additionally, the biocontrol potential of these isolates was evaluated by testing their antagonistic activity against *X. albilineans*, the causative agent of sugarcane leaf scald disease (Ntambo et al., 2019).

#### Narrow distribution of phosphate-solubilizing capacity among isolates

Phosphorus is an essential nutrient for plant growth and often exists in a form that is unavailable to plants (Shen et al., 2011). Phosphate-solubilizing bacteria play a crucial role in making phosphorus more bioavailable in soils, particularly in agricultural settings. Phosphate solubilization was tested using NBRIP solid medium, as described in Materials and methods. Out of the 32 isolates used in this work, only five demonstrated clear phosphate solubilization halos. In particular, three isolates from the rhizosphere (RB 214, 217, 258) and two isolates from the endosphere (EB 336, 340; Table 1). The fact that both RB and EB isolates showed phosphate solubilization activity aligns with previous research highlighting the crucial role of microorganisms in enhancing phosphorus availability in agricultural systems (Mitra et al., 2020). The Phosphate Solubilization Index (PSI) ranged from 1.1 to 1.4, with *Burkholderia* sp. RB 258 exhibiting the highest PSI (Table 1). Notably, the genera identified among the phosphate-solubilizing isolates are well-known for this function (Rodriguez and Fraga, 1999). Although the genus *Bacillus* is frequently reported as another major player in phosphate solubilization, our *Bacillus* isolates did not show this trait (Saeid et al., 2018). However, some studies have also reported *Bacillus* strains isolated from soil lacking phosphate solubilization ability (Sozer Bahadir et al., 2018).

#### Enhanced siderophore production among rhizospheric bacteria

Siderophores are molecules produced by bacteria that chelate iron from the environment, making it available to plants (Grobelak and Hiller, 2017). The siderophore production ability of our isolates was evaluated, and seven out of 32 isolates (five RB and two EB) exhibited this trait (Table 1). In line with previous reports, we found that both the number of producing isolates and the levels of siderophore production were higher among rhizospheric bacteria compared to endophytic ones (Abedinzadeh et al., 2019). Among RB isolates, RB 204, 208, 214, and 241 produced the largest halos while RB 258 showed levels comparable to those of the endophytic isolates EB336 and EB 344 (Table 1). These findings align with the well-established role of rhizospheric bacteria in siderophore production, which contributes to microbial competitiveness, facilitates plant–microbe signaling, and supports effective root colonization (Abedinzadeh et al., 2019).

#### IAA production reveals a stronger contribution from endophytic bacteria

IAA is a key phytohormone that regulates plant growth and development. Bacteria that produce IAA can enhance plant growth by stimulating root development and other physiological processes (Keswani et al., 2020). In this study, ten isolates were identified as IAA producers, of which, five corresponded to rhizospheric and five to endophytic isolates (Table 1). In particular, *Sphingobium* sp. EB 341 and *Chryseobacterium* sp. RB 216 being the highest producers among the endophytic and rhizospheric groups, respectively (Table 1). Interestingly, EB 341 was the isolate that showed the highest IAA production amongst all. This is in agreement with previous findings by Boss et al. (2022), who reported that this genus exhibited the highest IAA production among soil-derived isolates. Notably, endophytic isolates generally produced more IAA than rhizospheric isolates (Table 1). On the contrary, de Santi Ferrara et al. (2012) reported similar levels of IAA being released by both endophytic and rhizospheric sugarcane bacterial isolates. Even though these results suggest that this feature would not be affected by their location in the plant, the focus on solely enterobacterial isolates limits the conclusions to this family of microbes. Our results could be consistent with the understanding that endophytes might engage in more intricate interactions with the plant, potentially influencing plant growth more directly (Hardoim et al., 2015). Additionally, previous studies have inferred the importance of this hormone for endophytism, relating its effects on root development to a more efficient bacterial colonization (Abedinzadeh et al., 2019).

#### Biocontrol against *X. albilineans* restricted to rhizospheric isolates

The potential biocontrol activity of the isolates was tested *in vitro* against *X. albilineans*, the phytopathogen responsible for sugarcane leaf scald disease. Among the 32 isolates tested, four showed significant inhibitory activity against *X. albilineans*, and notably, all of them were RB (Table 1). This is somewhat surprising, given that transmission of the pathogen is generally characterized as mechanical due to wounds caused by knives and harvesters, and the pathogen is mostly associated with leaves and stalks (Daugrois et al., 2011). Although primarily considered a vascular pathogen, *X. albilineans* has been detected in roots and rhizosphere (Klett and Rott, 1994). However, evidence for a persistent, soil-borne or saprophytic “root cycle,” remains lacking. Two of the four isolates having antagonistic effects on *X. albilineans* belonged to genera already reported to inhibit *Xanthomonas* species (*Burkholderia* and *Enterobacter* strains; Guo et al., 2020; Alvarez et al., 2025). Although no reports to date have described *Chryseobacterium* or *Variovorax* strains with activity against *Xanthomonas*, both genera have been documented to exhibit biocontrol potential against other phytopathogens (Kim et al., 2012; Huang et al., 2017; Jose et al., 2019; Haedar et al., 2024). The fact that EB did not show an antagonistic effect on the pathogen, does not exclude endophytes protecting the plant through other mechanisms such as induced systemic resistance or impairment of virulence factor expression by inhibiting the quorum sensing system of the pathogen (de Santi Ferrara et al., 2012; Olanrewaju et al., 2017; Saeed et al., 2021). Finally, it remains to be elucidated whether the *in vitro* activity seen by RB is actually the result of a simply unspecific antagonistic effect natural to occur in a very competitive niche as the rhizosphere or it is somehow related to a poorly described root cycle of the pathogen (Maida et al., 2016; Hassani et al., 2018).

### Ecological interaction profiles of endophytic and rhizospheric communities

**Figure 2:**
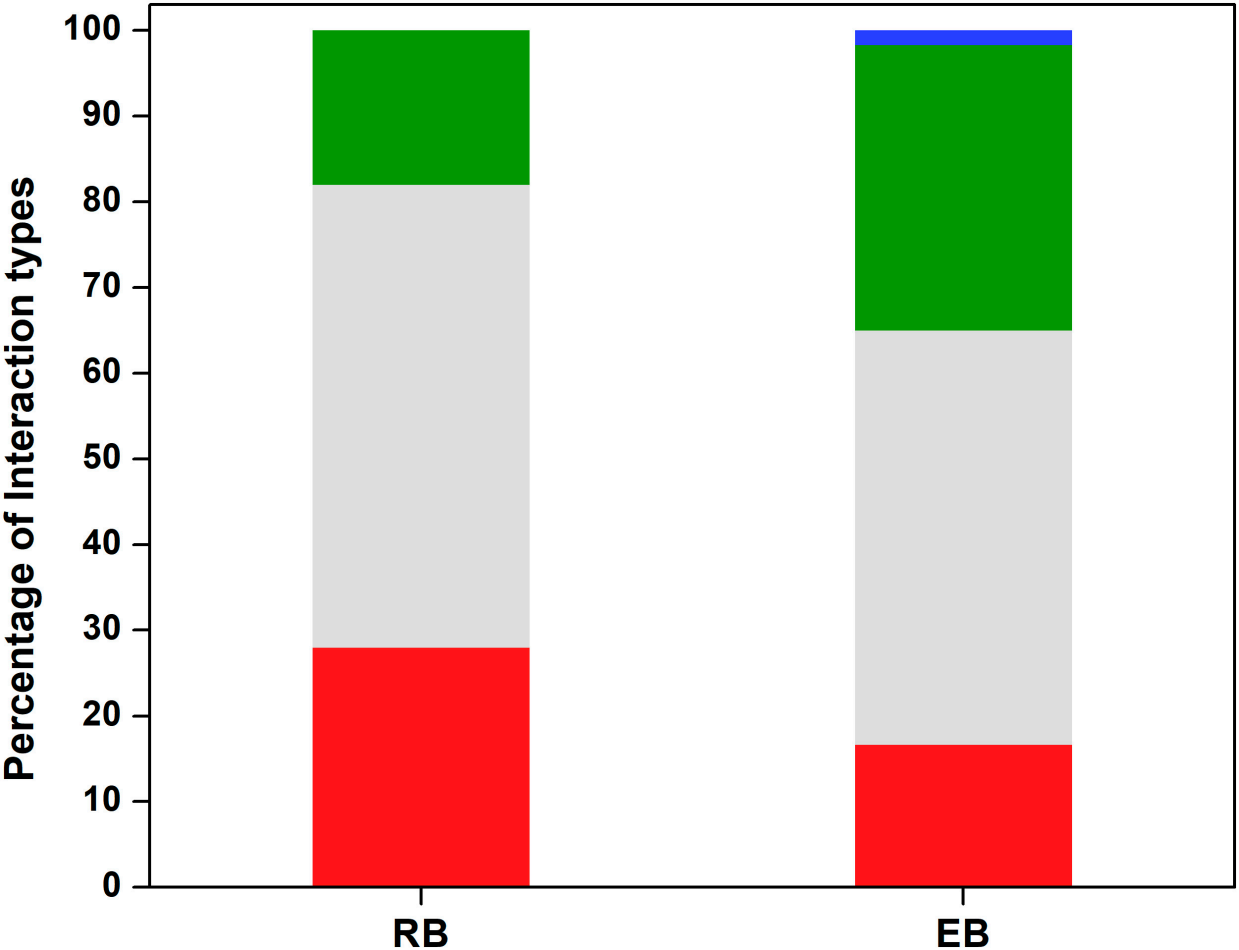
Classification of interaction outcomes among sugarcane-associated bacterial isolates. Percentage of pairwise interactions classified as positive (green), negative (red), neutral (gray), or singular (blue). Values are expressed as the percentage of total interactions observed in each group.

**Figure 3:**
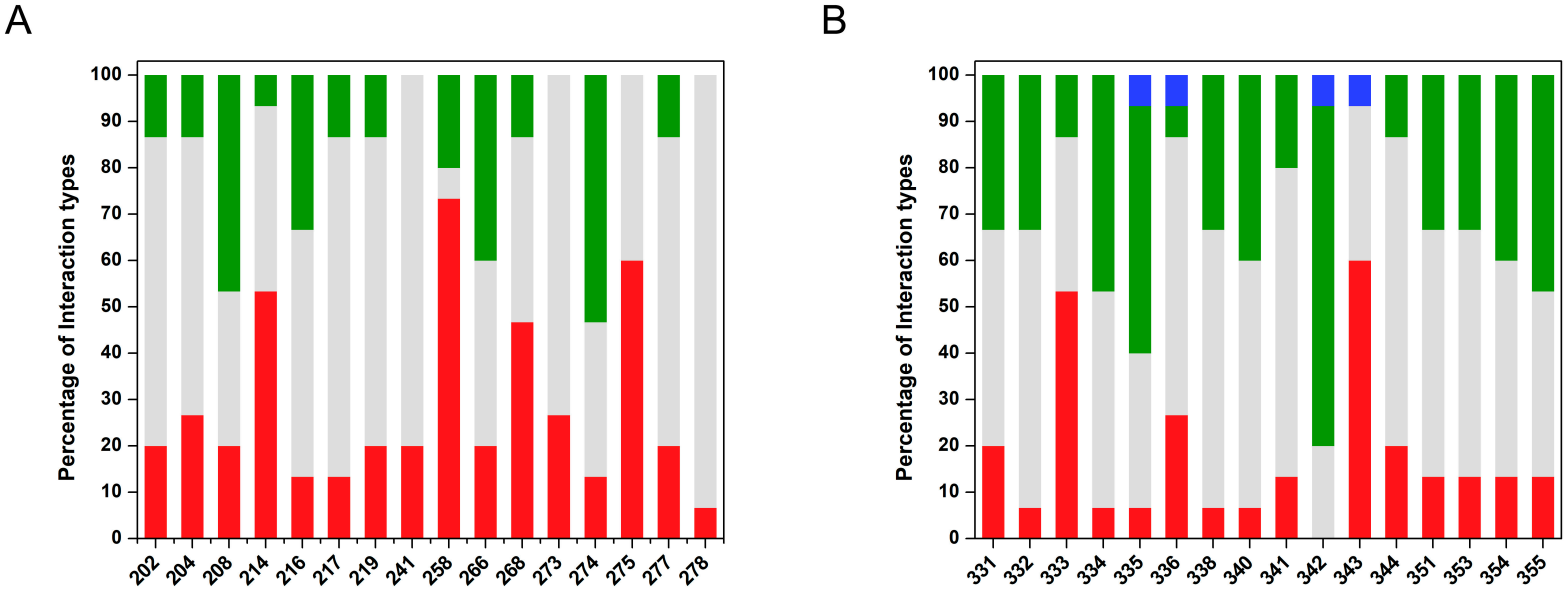
Individual interaction profiles of rhizospheric and endophytic isolates. Frequency of pairwise interactions involving each rhizospheric **(A)** and endophytic **(B)** isolate into positive (green), negative (red), neutral (gray), and singular (blue) categories. Percentages represent the proportion of interactions of each category relative to the total number of pairwise combinations for that isolate.

To investigate the potential for interactions between the isolates, we performed all possible pairwise combinations between the EB and RB isolates, respectively (120 interactions within each group). The analysis of pairwise interactions revealed that neutralism was the dominant interaction type across most bacterial isolates, comprising around 50% of the total interactions in both rhizospheric and endophytic communities (Figure 2). However, the distribution of positive and negative interactions varied between the two groups. RB isolates showed a higher proportion of negative interactions, while EB isolates exhibited a greater frequency of positive interactions (Figure 2). Although the predominance of negative interactions in microbial communities has been frequently reported in the literature (Foster and Bell, 2012; Ghoul and Mitri, 2016), recent findings suggest that positive interactions may be more common than previously thought (Kehe et al., 2021). Among RB isolates, we found that *Azospirillum* sp. RB 268, *Sphingomonas* sp. RB 275, *Burkholderia* sp. RB 258 and *Enterobacter* sp. RB 214 exhibited a remarkably high proportion of negative interactions (Figure 3A). Whereas, *Burkholderia* sp. RB 258 and *Enterobacter* sp. RB 214 displayed strong inhibitory effects on several isolates, *Azospirillum* sp. RB 268 and *Sphingomonas* sp. RB 275 were strongly inhibited by multiple strains (Supplementary Figure S2). In particular, isolate RB 258 showed the highest relative abundance of negative interactions, suggesting a potentially antagonistic role within the community (Figure 3A). This is consistent with previous reports showing that members of the *Burkholderia* genus produce a wide range of extracellular compounds, including toxins, antibiotics, siderophores, and enzymes, which contribute to their ecological versatility and competitive interactions with other microorganisms (Vial et al., 2007). Isolates such as *Arthrobacter* sp. RB 208 and *Microbacterium* sp. RB 274 displayed a greater frequency of positive interactions, indicating a possible beneficial influence on surrounding taxa (Figure 3A). Notably, studies have shown that *Arthrobacter* and *Microbacterium* are not only resilient members of the sugarcane rhizosphere microbiota but also contribute to plant growth promotion through multiple mechanisms (Pereira et al., 2019). Their frequent occurrence in positive ecological interactions suggests that these genera may play a key role in maintaining microbial community stability and supporting plant-associated functions. Curiously, *Pedobacter* sp. RB 278 was unique in that it did not exhibit any detectable effect on the growth of other isolates (Supplementary Figure S2). Unlike competitive interactions, which often require energetic investment and trade-offs, neutral coexistence can be considered a cost-effective strategy for surviving in complex microbial communities (Yan et al., 2021). It has been proposed that neutrality is more likely to emerge in species-rich systems, where the functional redundancy and high diversity may buffer the influence of any single taxon (Holt, 2006). Among the interactions observed within the EB isolates, we identified *singular* interactions. Specifically, we observed a negative effect at short distances and a positive effect at longer distances. For instance, *Pantoea* sp. EB 336 inhibited *Rothia* sp. EB 335 when grown in close proximity, yet promoted its growth at greater distances (Supplementary Figure S1B). A similar pattern was seen between *Microbacterium* sp. EB 342 and *Bacillus* sp. EB 343, where *Bacillus* exhibited distance-dependent antagonistic and beneficial effects on *Microbacterium* (Supplementary Figure S3). This phenomenon underscores the importance of spatial organization in shaping microbial interactions and highlights how the physical distance between cells can modulate the outcome of those interactions. In this sense, Romdhane et al. (2024) have demonstrated that manipulating intercellular distances can significantly influence microbial community assembly, emphasizing that not only the identity of microbial partners but also their physical arrangement plays a fundamental role in shaping microbial ecosystems. *Staphylococcus* sp. EB 333 and *Bacillus* sp. EB 343 showed a clear predominance of negative interactions (Figure 3B). Although *Staphylococcus* sp. EB 333 was involved in multiple negative interactions, a closer examination of the interaction matrix revealed that it was predominantly the target of inhibitory effects (Supplementary Figure S3). Notably, *Bacillus* sp. EB 343 inhibits the growth of other strains without being affected in return (Supplementary Figure S3). This is consistent with the known ability of *Bacillus* species to produce a broad spectrum of antimicrobial compounds, dedicating 5%−8% of their genome to the biosynthesis of secondary metabolites (Fira et al., 2018). On the other hand, *Microbacterium* sp. EB 342 exhibited the highest proportion of positive interactions, receiving beneficial effects from 12 out of 15 isolates (Figure 3B, Supplementary Figure S3). Positive bidirectional interactions consistently appeared in specific isolate pairs, such as EB 335 vs. EB 340, EB 342 vs. EB 332, EB 338 vs. EB 354, and EB 342 vs. EB 351, suggesting potential synergistic relationships between these strains (Supplementary Figure S3). When analyzing the interaction outcomes among isolates belonging to the same genus (e.g., *Acinetobacter* sp. EB 332, 338, 351, and 353; and *Brevundimonas* sp. EB 354 and 355), we observed no major differences when classifying interactions as positive, negative, or neutral (Figure 3B). However, a more detailed inspection of the interaction matrix revealed distinct patterns between strains of the same genus (Supplementary Figure S3). These differences suggest that, despite their taxonomic similarity, these isolates may correspond to different strains exhibiting phenotypic variation. While this study focused on macroscopically observable phenotypes, it is possible that molecular-level interactions occurred without visible manifestations, and these remain outside the scope of the present analysis.

### Microbial interactions reshape plant growth-promoting capacities

Although microbial interactions are known to influence the composition of plant-associated communities (Hassani et al., 2018), they may also modulate PGP traits in ways that are either beneficial or detrimental to the plant. Based on this premise, we analyzed the variation in PGP properties of isolates that originally displayed such traits when grown in interaction with other strains. While the present work does not explore the emergence of PGP functions resulting from microbial interactions, this remains an intriguing avenue for future research. In this regard, Tyc et al. (2014) previously described the induction of antibiotic production as a result of microbial interaction. To our knowledge, this is the first study to apply this type of interaction-based approach to evaluate PGP trait modulation. Regarding the biocontrol of *X. albilineans*, no change was observed in the *in vitro* activity against the pathogen in any of the interactions evaluated (Supplementary Figure S4).

#### Phosphate solubilization modulated by microbial partners

**Figure 4:**
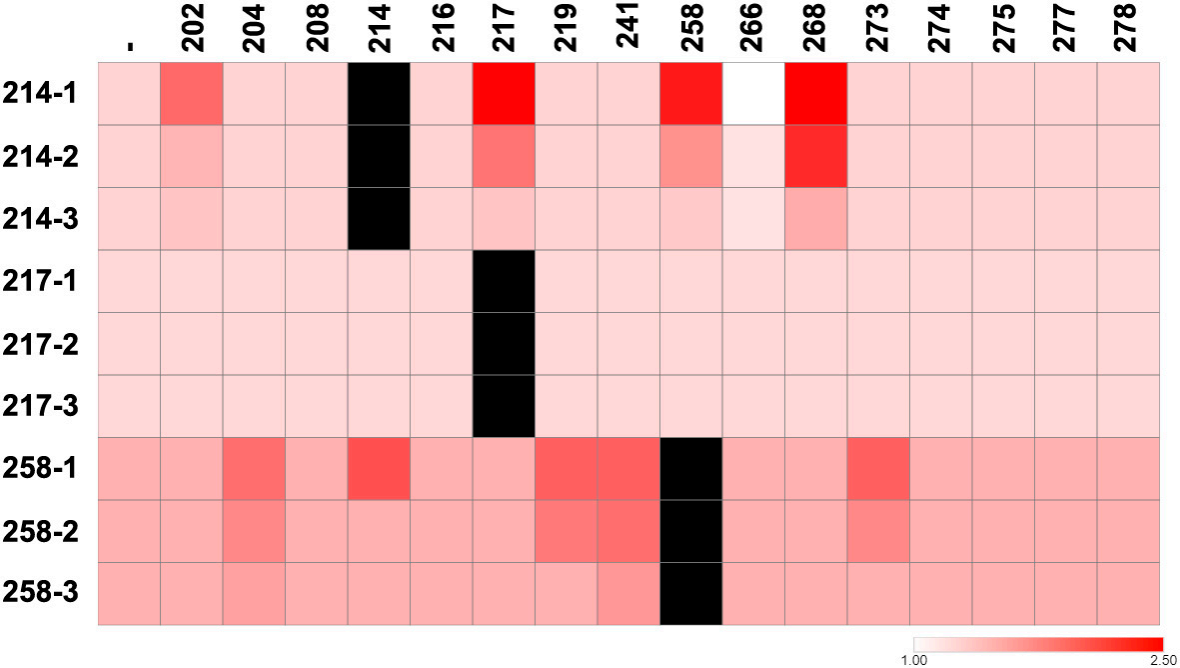
Effect of bacterial interactions on phosphate solubilization. Heatmap showing the phosphate solubilization capacity of bacterial isolates when interacting with other community members at three different distances: position 1 (closest proximity to the interacting partner), position 2, and position 3. Darker colors indicate higher solubilization levels. The first column (-) represents phosphate solubilization without interaction.

We further investigated how microbial interactions influenced the PSI of phosphate-solubilizing isolates. As mentioned above, *Burkholderia* sp. RB 258 was the isolate with the higher PSI (Table 1). As it can be seen in Figure 4, *Burkholderia* sp. RB 258 showed an increase in its PSI in the presence of RB 204, 214, 219, 241, and 273. As RB 204, 219, 241, and 273 do not solubilize phosphate, we assume that the increased phosphate solubilizing activity results from actual interactions. Among these isolates, three (RB 204, 219, and 273) showed a positive interaction with RB 258, while two (RB 214 and 241) exhibited neutral interactions (Supplementary Figure S2). Positive interactions with RB 258 most likely result in increased PSI due to enhanced bacterial proliferation. In the case of neutral interactions with RB 258, the mechanism underlying the increased PSI is less evident. It could imply complementary secretion of organic acids such as citric, oxalic, gluconic, lactic and others that acidify the environment and dissolve phosphate bound to cations (Zhang et al., 2025). Quorum sensing (QS), by regulating acid production, phosphate-solubilizing enzymes, and secondary metabolism has a key role in phosphate-solubilizing microbes (Garcia-Berumen et al., 2025). Then, it is possible that interactions that modulate QS activity have an impact on phosphate solubilization. Also, as siderophores can release phosphorus by binding Fe or Al that otherwise immobilize it, its production can certainly affect the PSI (Lei et al., 2025). Finally, exopolysaccharide production, by retaining water and acids and altering ion availability could also influence PSI (Luo et al., 2025). In the interaction with RB 214, another phosphate-solubilizing isolate, a marked increase was only observed at the closest distance, possibly reflecting an additive effect (Figure 4). Conversely, the interaction with RB 241 resulted in increased activity at all tested distances, with a decreasing trend as the distance increased (Figure 4). This spatial pattern suggests that diffusible metabolites released by RB 241 might be responsible for triggering phosphate solubilization in RB 258. *Enterobacter* sp. RB 214 exhibited an increased PSI when interacting with *Variovorax* sp. RB 202, *Pseudomonas* sp. RB 217, *Burkholderia* sp. RB 258, and *Azospirillum* sp. RB 268 (Figure 4). In contrast to its effect on RB 258, where the increase in PSI was observed only at close proximity, RB 214 showed enhanced phosphate solubilization at all tested distances when interacting with RB 258, with a gradual decline as the distance increased (Figure 4). This spatial pattern may be partially explained by the positive effect exerted by RB 258 on RB 214, which could stimulate metabolic activity (Supplementary Figure S2). Interestingly, *Azospirillum* sp. RB 268 induced a marked increase in the PSI of RB 214 despite maintaining a neutral interaction (Figure 4). Conversely, interaction with *Herbaspirillum* sp. RB 266 resulted in a notable reduction in PSI of RB 214, although interacting neutrally (Figure 4, Supplementary Figure S2). This reduction in PSI could be explained by consumption of organic acids by the non-solubilizing bacteria (Visser et al., 2025). On the other hand, PSI of *Pseudomonas* sp. RB 217 remained unaltered in the presence of the other isolates (Figure 4). These findings highlight the potential of interaction effects between rhizospheric bacteria that could lead to enhanced nutrient availability in the soil. Among EB, PSI of isolates capable of solubilizing phosphate was not significantly altered upon interaction with other isolates. This stability in activity suggests that the expression of phosphate-solubilizing traits in these bacteria may be less susceptible to modulation by neighboring taxa, at least under the experimental conditions tested.

#### Interaction-driven changes in siderophore production

**Figure 5:**
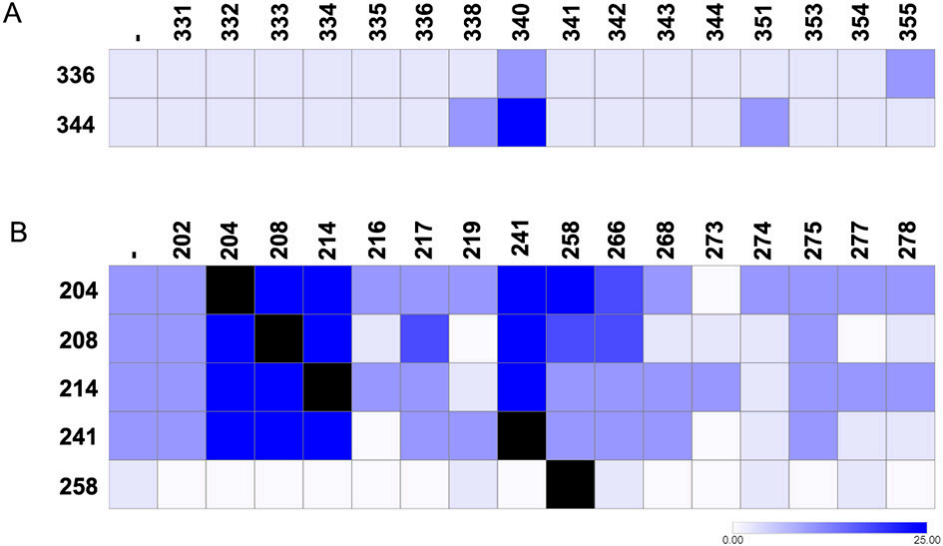
Effect of bacterial interactions on siderophore production. Heatmap showing the siderophore production capacity of bacterial isolates when interacting with other community members. **(A)** Endophytic isolates. **(B)** Rhizospheric isolates. Darker colors indicate higher siderophore production levels. The first column (-) represents siderophore production without interaction.

The impact of microbial interactions on siderophore production was also evaluated. As shown in Figure 5A, *Pantoea* sp. EB 336 exhibited increased siderophore production when exposed to *Pseudomonas* sp. EB 340 and *Brevundimonas* sp. EB 355. Interestingly, *Pantoea* sp. EB 336 inhibited the growth of *Pseudomonas* sp. EB 340, and considering previous reports indicating that siderophores can exert antimicrobial effects, it is plausible that siderophore production may contribute to this antagonistic interaction (Supplementary Figure S3; Choi et al., 2022). In the case of *Kocuria* sp. EB 344, siderophore production was increased by exposure to three isolates (EB 338, 340, and 351), despite the growth interactions being classified as neutral (Figure 5A, Supplementary Figure S3). The strongest induction was observed in response to *Pseudomonas* sp. EB 340 (Figure 5A). Notably, *Pseudomonas* sp. EB 340 was able to enhance siderophore production in both EB-producing strains, despite the fact that the growth interactions were classified as neutral, highlighting its potential role as an inducer of siderophore biosynthesis in neighboring strains (Figure 5A, Supplementary Figure S3). Moreover, when the two siderophore-producing strains were exposed to each other, no change in siderophore levels was observed, suggesting the absence of an additive effect (Figure 5A). These results suggest that certain isolates can function as inducers of siderophore production through mechanisms that operate independently of their growth interaction phenotype.

In contrast to what was observed for the EB isolates, several interactions led to a reduction in siderophore production by RB-producing strains (Figure 5B). Specifically, *Burkholderia* sp. RB 258 exhibited decreased siderophore production in the presence of eleven isolates (RB 202, 204, 208, 214, 216, 217, 241, 268, 273, 275, and 278; Figure 5B). None of these isolates inhibited RB 258; in fact, RB 258 exerted inhibitory effects on eight of them (Supplementary Figure S3). This indicates that the observed modulation in siderophore production is not a consequence of reduced growth of the producing isolate. Furthermore, siderophore production by *Arthrobacter* sp. RB 208 and *Rhizobium* sp. RB 241 decreased when exposed to seven (RB 216, 219, 268, 273, 274, 277, and 278) and five (RB 216, 273, 274, 277, and 278) isolates, respectively (Figure 5B). Notably, all interacting isolates exhibited a neutral interaction phenotype toward the siderophore producers, reinforcing the notion that modulation of siderophore production occurs independently of the growth interaction phenotype. According to Kramer et al. (2020), lower siderophore levels could be due to down regulation of production or siderophore piracy, but this remains to be elucidated for our interactions. The significant number of interactions resulting in reduced siderophore production suggest a probable general mechanism. On the other hand, isolates RB 204, 208, 214, 241 increased the siderophore production when interacting with each other (Figure 5B). This may be due to an additive effect or siderophore competition leading microbes to increase siderophore-mediated iron capture, as most interactions were phenotypically neutral (Supplementary Figure S2; Kramer et al., 2020).

#### Community interactions influence IAA production

**Figure 6:**
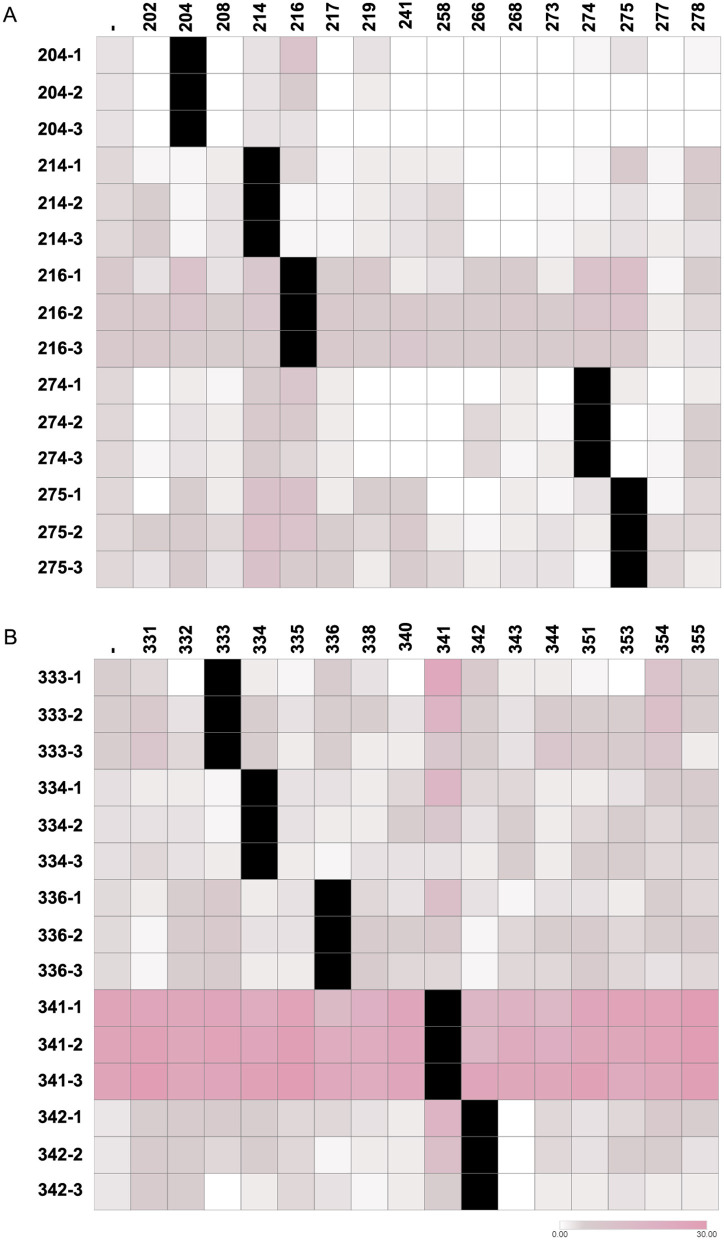
Effect of bacterial interactions on IAA production. Heatmap showing the IAA production capacity of bacterial isolates when interacting with other community members at three different distances: position 1 (closest proximity to the interacting partner), position 2, and position 3. **(A)** Rhizospheric isolates. **(B)** Endophytic isolates. Darker colors indicate higher IAA levels. The first column (-) represents IAA production without interaction.

Finally, we examined how microbial interactions affected IAA production. In line with previous reports, endophytic isolates tended to be stronger IAA producers. This trait is likely a key factor in their selection by plants, as it contributes to successful colonization and growth promotion (Etesami et al., 2014). Among the RB isolates, *Chryseobacterium* sp. RB 216 produced the highest amount of IAA, consistent with previous reports associating this genus with elevated IAA production (Figure 6A; Youseif, 2018; Chhetri et al., 2022). However, its production was modified due to interactions with other RB isolates (Figure 6A). RB 216 IAA production increased in the presence of isolates RB 204, 219, 268, 274, and 275, with stronger effects observed at positions closest to the interacting strain (Figure 6A). A decreased IAA production by *Chryseobacterium* sp. RB 216 was observed when interacting with isolates RB 202, 208, 217, 241, 258, 273, and 277 (Figure 6A). Interestingly, interaction of all RB IAA-producing isolates with *Flavobacterium* sp. RB 277 resulted in lower IAA levels (Figure 6A). Since RB 277 does not negatively affect the growth of these isolates (Supplementary Figure S2), we speculate that reduced IAA levels could either result from IAA degradation/metabolization or eventually downregulation of its synthesis. This capacity has been previously reported in other bacteria and may serve, among other purposes, the exploitation of IAA as a source of carbon, nitrogen, or energy (Laird et al., 2020). Regarding EB isolates, as mentioned above *Sphingobium* sp. EB 341 was the highest IAA producer among this group (Table 1). Interestingly, IAA production by *Sphingobium* sp. EB 341 decreased when interacting with EB 336, 342, and 344, whereas an increase was observed in the presence of EB 355, despite all these interactions being phenotypically neutral in terms of growth (Figure 6B, Supplementary Figure S3). It is important to note that IAA production increased for all isolates when interacting with EB 341; however, we suggest that this effect may result from IAA diffusion produced by EB 341 rather than an actual induction in IAA biosynthesis by the interacting isolates. In other case, IAA production by *Staphylococcus* sp. EB 333 increased in the presence of EB 354 and decreased when interacting with nine isolates (EB 331, 332, 334, 335, 340, 343, 344, 351, and 353; Figure 6B). Notably, six of these nine isolates inhibited the growth of EB 333, suggesting that the reduction in IAA production could be attributed, at least in part, to growth inhibition (Figure 6B, Supplementary Figure S3). The results suggest that IAA production is modulated by microbial interactions, which could influence the plant growth-promoting potential of these bacteria.

Taken together, our results suggest that functional traits were influenced by inter-bacterial interactions. Such modulation could have ecological relevance in shaping plant–microbe communication and microbiome functionality.

## Concluding remarks

This study provides a systematic characterization of how interspecies interactions among sugarcane-associated bacteria can modulate key PGP traits. Even with a relatively low numbers of isolates, we observed a wide diversity of interaction outcomes, including positive, negative, and neutral effects. These findings highlight that microbial behavior cannot be fully predicted from monocultures and that interaction-driven modulation is a fundamental layer of plant–microbe functionality. Notably, some effects were distance-dependent, illustrating the influence of spatial structure within microbial communities. Importantly, functional modulation was detected even when growth phenotypes remained unchanged, underscoring the relevance of hidden molecular interactions. Although not explicitly assessed here, microbial interactions may lead to the emergence of “hidden” or conditionally expressed functions, whereby strains that do not exhibit a given trait in monoculture could express it when interacting with a partner (Tyc et al., 2014; Abrudan et al., 2015).

Together, these results emphasize the value of interaction-based screening as a first step to identify promising strains and consortia with agronomic potential. While our *in vitro* assays allowed the systematic exploration of interaction patterns, determining their ecological significance requires *in planta* validation. Building on the interaction phenotypes described here, future studies will evaluate the most relevant isolates and combinations under greenhouse and field conditions, and will incorporate complementary approaches such as transcriptomics, metabolomics, and quantitative chemical assays for siderophore and IAA quantification. Such efforts will help uncover the mechanistic basis of interaction-driven functional shifts and determine their contribution to plant growth promotion.

## Data Availability

The datasets presented in this study can be found in online repositories. The names of the repository/repositories and accession number(s) can be found in the article/Supplementary material.
