# Supplementary material for: Interspecies interactions among sugarcane-associated bacteria and their impact on plant growth promotion traits
Source: Front Microbiol. 2026 Jan 29;17:1714037. doi: 10.3389/fmicb.2026.1714037 (PMC12894300; doi:10.3389/fmicb.2026.1714037)
Supplement: Supplementary file 1 [file Data_Sheet_1.pdf]

Figure S1

A)

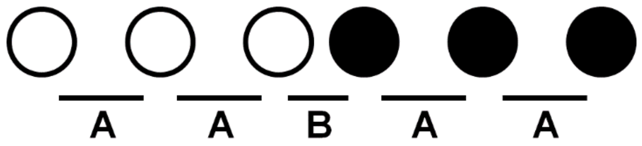

B)

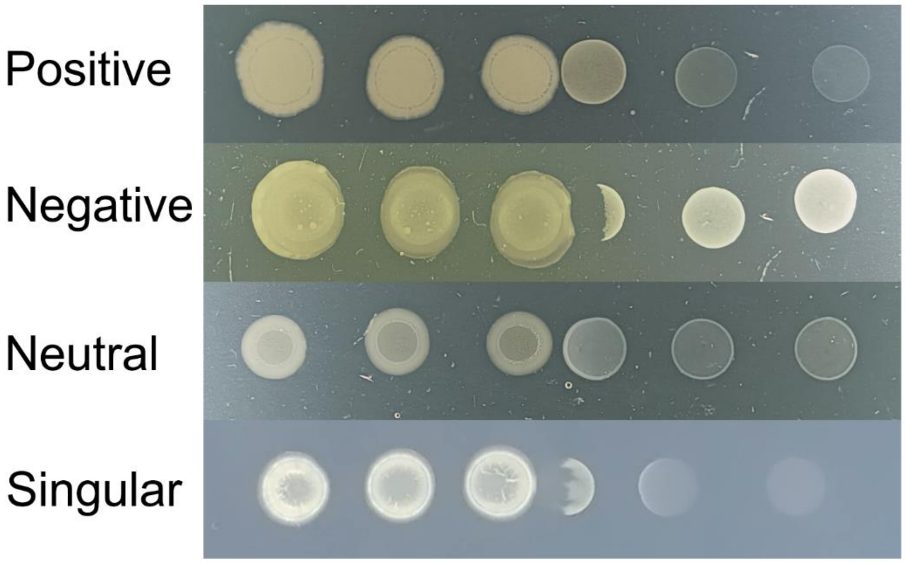

Figure S1. A) Distance pattern used for binary interactions assays. Each color spot corresponds to two different isolates. A corresponds to 1 cm distance between spots, and B a 0,5cm distance between different isolates spots. **B)** Representative examples of each interaction outcome.

Figure S2

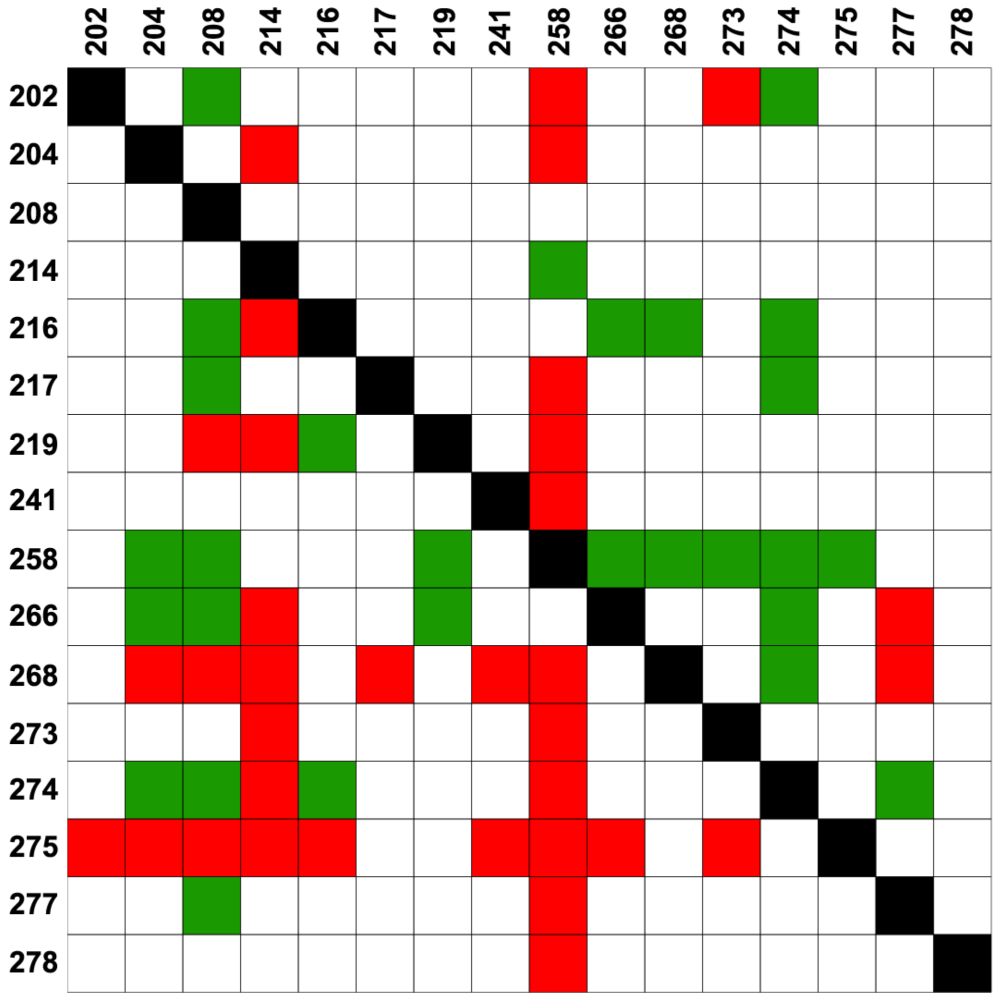

Figure S2. RB isolates interaction matrix. Interaction outcomes were categorized as positive (green), negative (red), or neutral (white). Each square represents the effect exerted by the isolate listed in the column on the growth of the isolate indicated in the row. For instance, RB 202 showed no visible change when exposed to RB 204 (white square), whereas its growth was stimulated in the presence of RB 208 (green square).

Figure S3

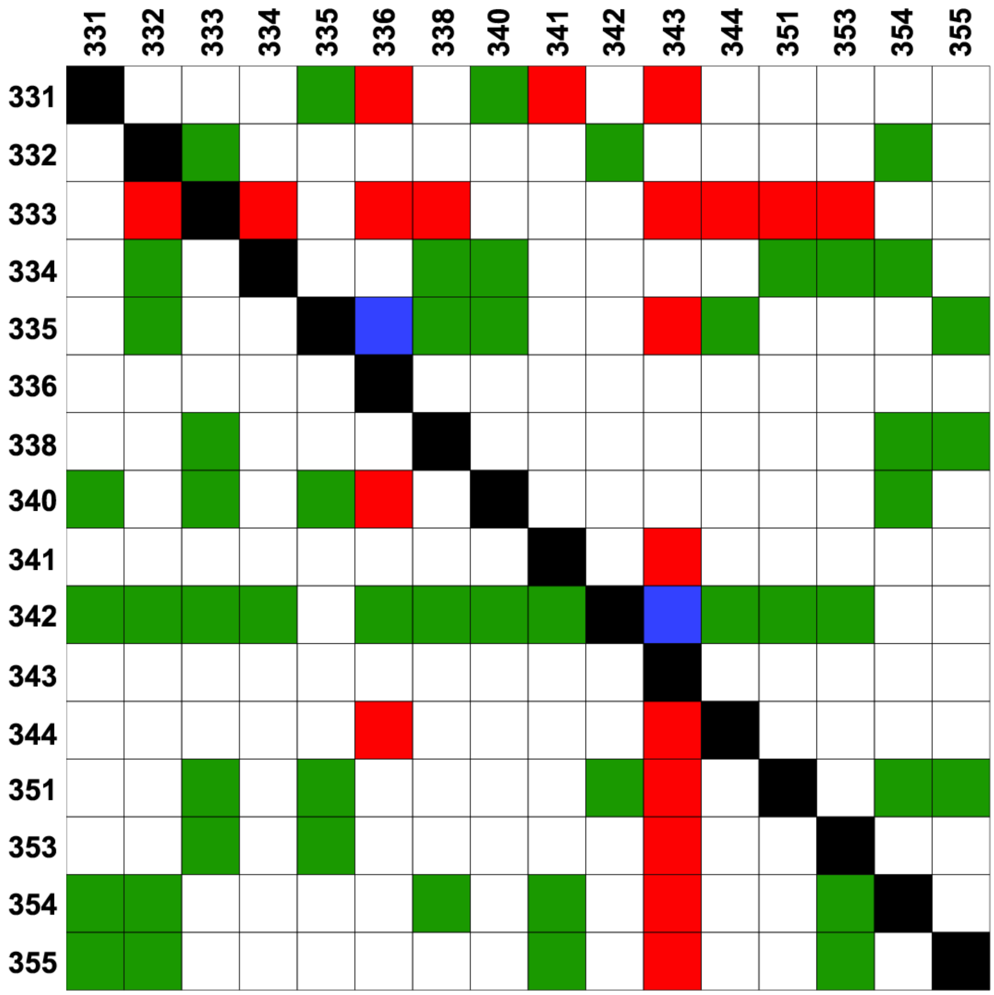

Figure S3. EB isolates interaction matrix. Interaction outcomes were categorized as positive (green), negative (red), neutral (white), or singular (blue). Each square represents the effect exerted by the isolate listed in the column on the growth of the isolate indicated in the row. For instance, EB 340 was inhibited when exposed to EB 336 (red square), whereas its growth was stimulated in the presence of EB 335 (green square).

Figure S4

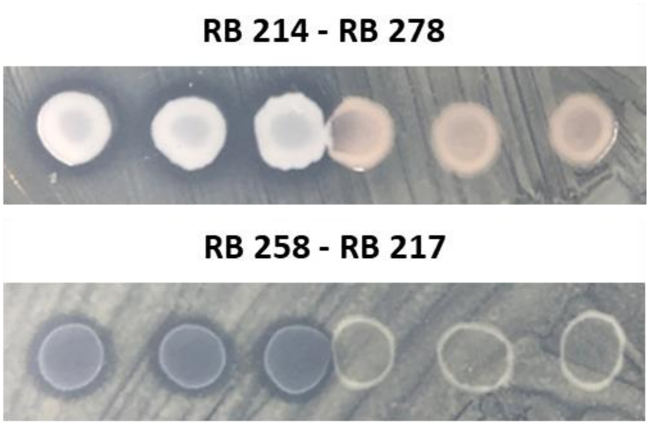

Figure S4. Representative examples of pairwise interactions that did not alter antagonistic activity against *Xanthomonas albilineans*. For each pair, both isolates retained their original antagonistic phenotype, indicating that the evaluated interactions did not influence biocontrol activity.

Table S1. Details of the classification of isolates with the least common ancestor (LCA) method, based on the taxonomies hosted by SILVA, after the alignment of 16S rRNA sequences with SILVA ACT (https://www.arb-silva.de/aligner). Shown are the score, percent identity and quality of the alignments, the length of the sequences (in bp), and the corresponding taxonomy of the isolates.

strain	score	% id.	quality	bp	taxonomy-silva
RB202	0.999016	99.8534	99	1364	Gammaproteobacteria;Burkholderiales;Comamonadaceae;Variovorax;
RB204	0.997982	99.9294	99	1416	Bacilli;Bacillales;Bacillaceae;Bacillus;
RB208	0.999208	98.6192	99	1373	Actinobacteria;Micrococcales;Micrococcaceae;Arthrobacter;
RB214	0.996663	99.7514	99	1207	Gammaproteobacteria;Enterobacterales;Enterobacteriaceae;Enterobacter;
RB216	0.997805	99.3926	99	1314	Bacteroidia;Flavobacteriales;Weeksellaceae;Chryseobacterium;
RB217	0.993967	98.984	99	1378	Gammaproteobacteria;Pseudomonadales;Pseudomonadaceae;Pseudomonas;
RB219	0.964944	97.6628	96	1223	Actinobacteria;Micrococcales;Microbacteriaceae;Curtobacterium;
RB241	0.997496	99.8241	99	1137	Alphaproteobacteria;Hyphomicrobiales;Rhizobiaceae;Rhizobium;
RB258	0	100	100	1272	Gammaproteobacteria;Burkholderiales;Burkholderiaceae;Burkholderia-Caballeronia-Paraburkholderia;
RB266	0.997982	99.8555	99	1383	Gammaproteobacteria;Burkholderiales;Oxalobacteraceae;Herbaspirillum;
RB268	0.998574	98.133	99	853	Alphaproteobacteria;Azospirillales;Azospirillaceae;Azospirillum;
RB273	0.993717	98.7407	99	1346	Alphaproteobacteria;Sphingomonadales;Sphingomonadaceae;Novosphingobium;
RB274	0.999057	99.8521	99	1350	Actinobacteria;Micrococcales;Microbacteriaceae;Microbacterium;
RB275	0.999	99.1045	99	1339	Alphaproteobacteria;Sphingomonadales;Sphingomonadaceae;Sphingomonas;
RB277	0.99442	98.2315	99	622	Bacteroidia;Flavobacteriales;Flavobacteriaceae;Flavobacterium;
RB278	0.999242	99.2526	99	1338	Bacteroidia;Sphingobacteriales;Sphingobacteriaceae;Pedobacter;
EB331	0.994668	99.1189	99	1359	Alphaproteobacteria;Sphingomonadales;Sphingomonadaceae;Sphingomonas;
EB332	0.997941	99.7169	99	1410	Gammaproteobacteria;Pseudomonadales;Moraxellaceae;Acinetobacter;
EB333	0.997036	99.9274	99	1430	Bacilli;Staphylococcales;Staphylococcaceae;Staphylococcus;
EB334	0.996043	99.8521	99	1352	Alphaproteobacteria;Hyphomicrobiales;Rhizobiaceae;Agrobacterium;
EB335	0.999054	98.638	99	1388	Actinobacteria;Micrococcales;Micrococcaceae;Rothia;
EB336	0.996587	99.8515	99	1522	Gammaproteobacteria;Enterobacterales;Erwiniaceae;Pantoea;
EB338	0.997885	99.5007	99	1398	Gammaproteobacteria;Pseudomonadales;Moraxellaceae;Acinetobacter;
EB340	0	100	100	1380	Gammaproteobacteria;Pseudomonadales;Pseudomonadaceae;Pseudomonas;
EB341	0.999001	99.8488	99	1323	Alphaproteobacteria;Sphingomonadales;Sphingomonadaceae;Sphingobium;
EB342	0.996849	99.2366	99	1386	Actinobacteria;Micrococcales;Microbacteriaceae;Microbacterium;
EB343	0	100	100	1411	Bacilli;Bacillales;Bacillaceae;Bacillus;
EB344	0.998635	99.2012	99	1376	Actinobacteria;Micrococcales;Micrococcaceae;Kocuria;
EB351	0.994939	99.6828	99	1334	Gammaproteobacteria;Pseudomonadales;Moraxellaceae;Acinetobacter;
EB353	0.999113	99.8558	99	1386	Gammaproteobacteria;Pseudomonadales;Moraxellaceae;Acinetobacter;
EB354	0.997743	99.2413	99	1316	Alphaproteobacteria;Caulobacterales;Caulobacteraceae;Brevundimonas;
EB355	0.997764	99.1704	99	1323	Alphaproteobacteria;Caulobacterales;Caulobacteraceae;Brevundimonas;
